# How does solid feed composition affect reticular development in calves receiving high milk replacer? A comparison of starter concentrate and alfalfa hay

**DOI:** 10.29374/2527-2179.bjvm010825

**Published:** 2026-04-30

**Authors:** Noelia Vazquez, Dellis Dos Santos, Germán Antúnez, Rody Artigas, Nicolás Amaro, Flavia Zanolli, Emilia Lanza, Cecilia Cajarville

**Affiliations:** 1 Departamento de Biociencias, Unidad de Anatomía, Facultad de Veterinaria, Universidad de la República, Montevideo, Uruguay.; 2 Departamento de Ciencias Veterinarias y Agrarias (CENUR-LN), Facultad de Veterinaria, Universidad de la República, EEMAC, Paysandú, Uruguay.; 3 Departamento de Producción Animal y Salud de los Sistemas Productivos, Unidad de Mejora Animal, Facultad de Veterinaria, Universidad de la República, Montevideo, Uruguay.; 4 Departamento de Producción Animal y Salud de los Sistemas Productivos, Facultad de Veterinaria, Universidad de la República, Libertad, San José, Uruguay.; 5 Veterinario autónomo, Montevideo, Uruguay.; 6 Departamento de Producción Animal y Salud de los Sistemas Productivos, Facultad de Veterinaria, Universidad de la República, Libertad, San José, Uruguay.

**Keywords:** calf, forestomach, reticulum, reticular crests, bezerro, cristas reticulares, pré-estômago, retículo

## Abstract

Reticulum, the second compartment of the ruminant stomach, is crucial in the stratification and sorting of ingested particles. Early-life nutrition influences forestomach development; however, its specific effects on reticular morphology are not well understood. This study evaluated the impact of two solid feed regimens—starter concentrate (Group A) versus alfalfa hay (Group B)—on reticular development in 60-day-old Holstein calves receiving 8 L/day of milk replacer. Twenty male calves were randomly assigned to the dietary treatments, and feed intake, body weight, and reticular morphometry were assessed. Reticulum samples were examined macroscopically, histologically, and using scanning electron microscopy. No significant differences were found in gross reticulum weight or linear dimensions between groups. Histological analyses, however, revealed greater structural complexity in Group B. Forage-fed calves exhibited more developed primary, secondary, and tertiary reticular crests (*cristae reticulares*), larger papillae along free margins, and more prominent unguiculiform papillae. These structural features likely improve mechanical sorting and retention of fibrous particles, promoting efficient fermentation. Starter-fed calves showed simpler crest architecture and smaller papillae, consistent with lower fiber intake; however, papillary development was still evident, indicating that solid feed contributes to epithelial growth. These findings emphasize the reticulum as a distinct anatomical and functional unit within the forestomach. Early inclusion of adequate dietary fiber through alfalfa hay enhances reticular complexity, which may support more effective particle sorting, improved fermentation, and long-term digestive efficiency. Overall, pre-weaning feeding strategies that incorporate fiber can positively influence reticular development, with potential benefits for nutrient absorption, digestive function, and calf growth.

## Resumo

O retículo, o segundo compartimento do estômago de ruminantes, desempenha um papel fundamental na estratificação e separação das partículas ingeridas. A nutrição na fase inicial da vida influencia o desenvolvimento do rúmen e do retículo, mas os efeitos específicos sobre a morfologia reticular ainda não são totalmente compreendidos. Este estudo avaliou o impacto de dois regimes de alimentação sólida—concentrado inicial (Grupo A) versus feno de alfafa (Grupo B)—no desenvolvimento reticular de bezerros Holandeses de 60 dias recebendo 8 L/dia de substituto lácteo. Vinte bezerros machos foram distribuídos aleatoriamente entre os tratamentos, e foram avaliados consumo de alimento, peso corporal e morfometria reticular. Amostras do retículo foram analisadas macroscópica, histológica e por microscopia eletrônica de varredura. Não foram observadas diferenças significativas no peso total ou nas dimensões lineares do retículo entre os grupos. Entretanto, análises histológicas revelaram maior complexidade estrutural no Grupo B. Bezerros alimentados com forragem apresentaram cristas reticulares (*cristae reticulares*) primárias, secundárias e terciárias mais desenvolvidas, papilas maiores nas margens livres e papilas unguiculiformes mais proeminentes. Essas características estruturais provavelmente melhoram a retenção e separação mecânica das partículas fibrosas, promovendo fermentação eficiente. Bezerros alimentados com concentrado apresentaram arquitetura de cristas mais simples e papilas menores, condizente com menor ingestão de fibra, embora o desenvolvimento papilar ainda fosse evidente, indicando que o alimento sólido contribui para o crescimento epitelial. Estes achados destacam a importância de considerar o retículo como uma unidade anatômica e funcional distinta dentro do pré-estômago. A inclusão precoce de fibra dietética adequada via feno de alfafa aumenta a complexidade reticular, potencialmente favorecendo a separação eficiente de partículas, melhor fermentação e maior eficiência digestiva a longo prazo. Estratégias de alimentação pré-desmame que incorporam fibra podem influenciar positivamente o desenvolvimento reticular, com benefícios potenciais para absorção de nutrientes, função digestiva e crescimento dos bezerros.

## Introduction

Reticulum, the second compartment of the ruminant stomach, plays a pivotal role in the stratification and sorting of ruminal contents. Through coordinated contractions, it redirects larger, buoyant particles back to the rumen, while finer, denser particles and fluids are selectively transferred to the omasum for subsequent stages of digestion (Clauss et al., 2009).

Reticular internal morphology varies markedly among ruminant species, particularly in the height of the reticular crests (*cristae reticulares*) and depth of the polygonal cells. Grazing species, characterized by a predominantly grass-based diet, typically exhibit more pronounced and intricate reticular crests—often incorporating secondary, tertiary, and even quaternary subdivisions—compared with browsing species (Hofmann, 1973; Langer, 1988).

Evidence presented by Clauss et al. (2009) demonstrated a significant positive association between crest height and the proportion of grass in the natural diet, although no consistent relationship was observed between crest height and the overall dimensions of the reticulum (height and width). Additionally, grazers generally possess a smaller reticulo-omasal orifice than browsers, a feature that likely contributes to slower digesta transit and allows for prolonged microbial fermentation of cellulose.

Unguiculiform papillae constitute another anatomically relevant feature, with their occurrence and degree of development varying across species. These structures have been proposed to function as a physical filtration mechanism, limiting the passage of coarse particles to the omasum (Teixeira et al., 2009).

During early rearing, nutritional management plays a decisive role in shaping the morpho-functional development of the young ruminant forestomach. High volumes of milk intake (8 L/day) are often associated with reduced solid feed consumption, which can delay the maturation of gastric compartments (Jasper & Weary, 2002). In a preceding study from the same experiment, no substantial differences were observed in abomasal morphology between calves fed the two dietary supplements (Vazquez et al., 2025); however, significant differences were found in the rumen (Vazquez et al., 2026), and similar differences are expected to be observed in the reticulum.

The present study investigated reticular development in 60-day-old Holstein calves under two feeding regimes: (A) milk replacer (MR) supplemented with starter feed, and (B) milk replacer supplemented with alfalfa hay. The primary objectives were to determine whether reduced fiber intake during early life influences reticular morphology and assess potential differences between dietary groups. Based on previous reports, we hypothesized that calves receiving milk replacer and forage would exhibit more advanced development of the reticulum and its crests compared with calves receiving concentrate as the primary solid feed.

## Materials and methods

This study was conducted at the Instituto de Producción Animal, Facultad de Veterinaria, Universidad de la República (UdelaR, route 1, km 42, Libertad, San José, Uruguay; 34º40’40’’S, 56º32’13’’W). Following the same experimental conditions described by Vazquez et al. (2025, 2026), twenty newborn male Holstein calves with adequate passive transfer of immunity were individually housed and fed 8 L/day of milk replacer until weaning. Animals were randomly assigned to two groups: Group A (starter concentrate; mean initial BW 41.4 ± 2.2 kg) or Group B (alfalfa hay; 39.1 ± 4.2 kg). Beginning in week 8, calves were gradually weaned, and from weeks 9–10 they had access to both starter and hay.

### Feed intake and growth

Feed analysis followed the procedures of AOAC (Association of Analytical Communities, 2000) and Van Soest et al. (1991) (Table 1). Daily intake was recorded, body weight (BW) was measured weekly, and feed efficiency was calculated as corrected BW gain relative to total feed intake.

**Table 1:** Chemical composition of milk replacer, starter feed, and alfalfa hay.

**Variables**	**Milk replacer**	**Starter feed**	**Alfalfa hay**
DM (%)	95.3	90.0	90.5
Crude Protein (% DM)	21.5	18.0	16.0
NDF (% DM)	−	17.9	40.4
ADF (% DM)	−	7.2	32.4
Ash (% DM)	5.6	5.6	7.2
Ether extract (EE) (% DM)	20.0	3.4	-
ME (Mcal/kg DM) 1	4.58	3.00	1.96
Aflatoxins B1, B2, G1, G2 (ppb)*	-	< 5	-
DON (ppb)*	-	< 500	-
Zearalenone (ppb)*	-	< 50	-

The variables indicated with * correspond to values reported on the product label.

Dry matter (DM), Neutral Detergent Fiber (NDF), Acid Detergent Fiber (ADF), Deoxynivalenol (DON).

1 Metabolizable energy (ME) was calculated according to Drackley (2008) for milk replacer, and according to NRC (National Research Council, 2001) for concentrate and alfalfa hay;

Lactose was estimated as: DM-(CP+EE+Ash).

Starter feed concentrate (per kg of DM) contained: 0.8–1.1% Ca, 0.7–0.9% P, 4,800 IU vitamin A, 600 IU vitamin D3, 120 mg vitamin E, 28.8 mg vitamin B1, 7.2 mg biotin, 5,760 mg Cu, 18,000 mg Mn, 0.228 mg I, 18,000 mg Zn, 0.062 mg Co, 0.115 mg Se, 16,800 mg Fe, and 30 mg of monensin.

### Euthanasia and anatomical evaluation

At 10 weeks, calves were euthanized following procedures reviewed and approved by the Animal Use Ethics Committee (Comisión de Ética de Uso de Animales; CEUA protocol Nº685) of Facultad de Veterinaria, UdelaR.. The reticulum was dissected, weighed (full and empty), and its dimensions recorded. Crest height was determined by measuring five representative cells per reticular region (proximal to the cardia, mid-region, and near the omasum), and the mean value was calculated. In addition, the length and thickness of the reticular groove lips were obtained from cross-sections of formalin-fixed tissue.

### Sample collection and microscopy

Tissue samples (1 × 1 cm) from six reticular regions—proximal to the rumen, mid-reticulum, reticulo-omasal orifice (*ostium reticulo-omasale*), unguiculiform papillae, reticular lips (*labia sulci reticuli*), and depth of the reticular groove (*sulcus reticuli*)—were collected from two animals per group. For scanning electron microscopy (SEM), samples were processed and imaged using a Jeol JSM 5900 LV at Facultad de Ciencias, UdelaR. For histological analysis, samples were paraffin-embedded, sectioned, and stained with Masson-Goldner trichrome at the Universidade Federal de Santa Maria (Brazil).

### Statistical analysis

Data normality was verified using the Shapiro–Wilk test. Independent t-tests and generalized linear mixed models (GLIMMIX, SAS) were used, with treatment as a fixed effect and initial BW as a covariate. Statistical significance was set at P ≤ 0.05.

## Results

### Food consumption and performance

During the experimental period, milk replacer intake was similar between both groups. During the first nine weeks, calves in Group A consumed on average 55.3% of dry matter (DM) from milk replacer and 44.7% from starter concentrate. In contrast, calves in Group B consumed 73.2% of DM from milk replacer and 26.8% from alfalfa hay.

Over the 10-week study period, calves in Group A consumed significantly greater amounts of total solid feed and total dry matter (p < 0.01), as well as more starter concentrate (p < 0.01), while their alfalfa intake was lower (p < 0.01) compared with Group B.

No significant differences were observed between groups in final body weight, total weight gain, or feed efficiency (Table 2).

**Table 2:** Total feed ingestion and performance throughout the 10-week experiment.

**Item**	**Treatments** 1		**SEM**	***P*-value** 2
	**Alfalfa hay**	**Starter feed**		
**Feed ingestion (wk 1-10 of life)**				
Total MR intake, kg DM	46.4	46.7	0.52	0.915
Total starter DM intake, kg	14.4	37.8	3.11	< 0.01
Total alfalfa hay DM intake, kg	17.0	5.7	1.56	< 0.01
Total solid feed DM, 3 kg DM	31.2	43.3	3.41	< 0.01
Total DM intake, 4 kg	77.6	90	7.10	< 0.01
Proportion of forage, 5^%^	54.5	13.2	2.76	< 0.01
Forage/Concentrate, %	1.18	0.15		< 0.01
**Performance (wk 1-10 of life)**				
Final BW, 6 kg	77.0	80.5	3.09	0.246
Total BW gained, ^6^ kg	36.9	40.3	3.08	0.246
Gain-to-feed, 7^%^	34.8	31.1	2.65	0.229

1 Treatments: The animals received 8 liters of milk replacer (MR) per day, free access to alfalfa hay or starter feed as solid feed before weaning (wk 8), and free access to alfalfa hay and starter feed from week 9 to 10 of the study;

2 Effect of treatment;

3 Total solid feed intake: sum of alfalfa hay and starter feed intake;

4 As the sum of dry matter (DM) intake from MR, alfalfa hay, and starter feed;

5 Forage intake as a proportion of the sum of concentrate and forage consumed;

6 Adjusted for gut fill similarly to Antúnez-Tort et al. (2023);

7 As the difference between final and initial body weight (BW) corrected for gut fill, divided by total feed intake.

### Macroscopic morphometry

**Figure 1:**
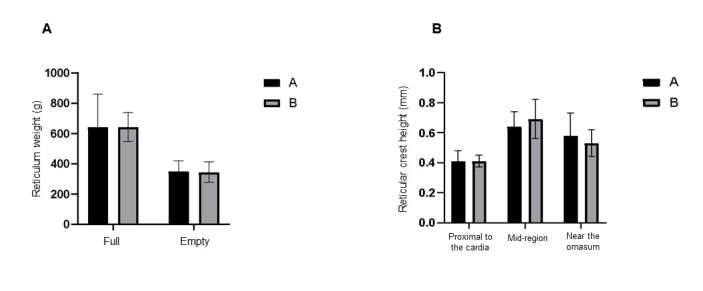
Comparison of results between Holstein calves fed 8 L/day milk replacer with starter (A) or alfalfa hay (B); data shown as mean ± SD. A- Full and empty reticulum weight. B- Reticular crest height.

The empty reticulum represented 0.38% of body weight in calves from Group A (starter) and 0.39% in calves from Group B (alfalfa). When full reticulum weight was considered, values were 0.70% and 0.73% of body weight, respectively. The weights of the full and empty reticulum are presented in Figure 1.

Morphometric measurements of the reticulum are summarized in Table 3. No statistically significant differences were observed between dietary groups for any evaluated parameter, including reticulum height, length, or crest dimensions.

**Table 3:** Mean ± standard deviation (SD) of reticular morphometric parameters in 60-day-old Holstein calves fed 8 L/day of milk replacer, water *ad libitum*, and either forage or concentrate *ad libitum*. Results of Student’s *t*-test.

**Parameter**	**Forage (Mean ± SD)**	**Concentrate (Mean ± SD)**	***p* value**
Calf body weight before slaughter (kg)	87.75 ± 7.66	91.90 ± 8.72	–
Reticulum full weight (g)	641.89 ± 96.61	642.50 ± 217.40	0.994
Reticulum height (cm)	14.44 ± 1.94	14.11 ± 1.45	0.686
Reticulum length (cm)	17.00 ± 2.00	16.44 ± 2.46	0.606
Reticulum empty weight (g)	343.07 ± 67.85	349.28 ± 68.81	0.931
Thickness of right reticular groove lip (mm)	21.17 ± 4.67	24.88 ± 8.87	0.373
Thickness of left reticular groove lip (mm)	20.00 ± 3.16	21.13 ± 6.60	0.708
Height of the reticular ridges proximal to the cardias	0.41 ± 0.07	0.41 ± 0.04	1
Height of the reticular ridges mid region	0.64 ± 0.1	0.69 ± 0.13	0.471
Height of the reticular ridges near the omasum	0.58 ± 0.15	0.53 ± 0.09	0.590

Mean primary crest height, averaged across the three reticular regions (cardia-adjacent, mid-reticulum, and near the omasum), did not differ significantly between groups (Figure 1, Table 3).

### Regional characteristics of the reticulum

#### General organization of the mucosa

**Figure 2:**
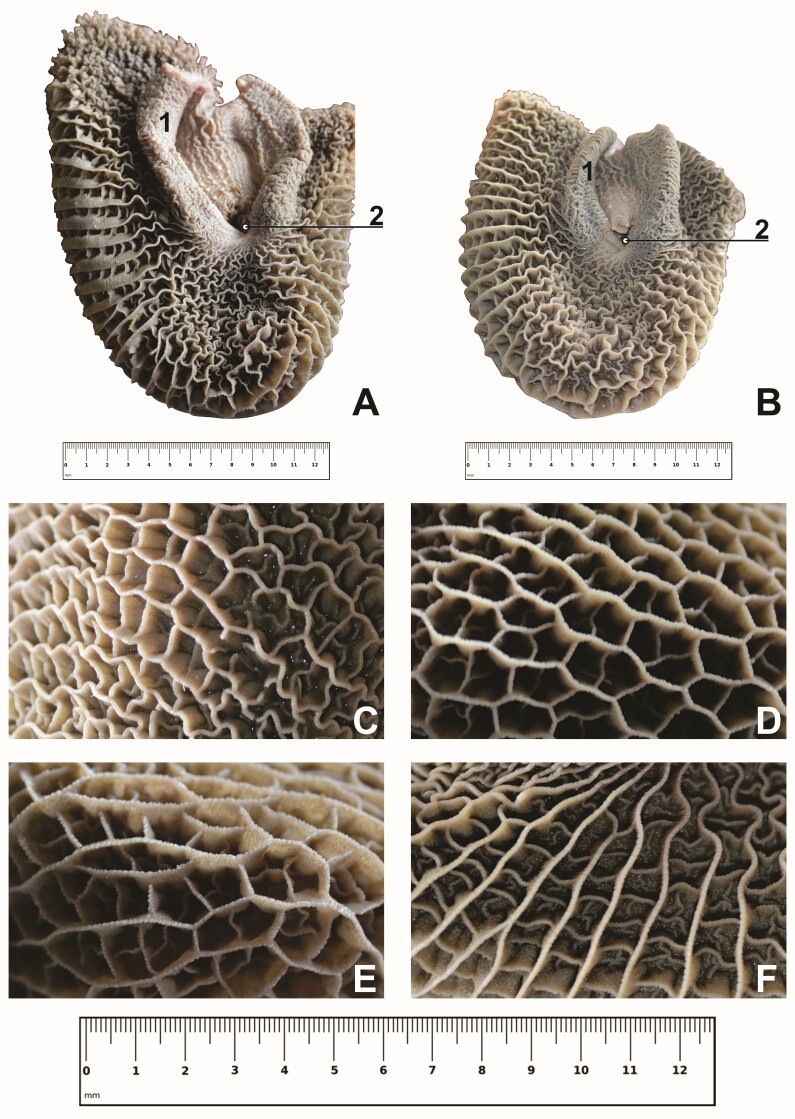
Macroscopic images of the reticulum illustrating reticular cell development. Images on the left correspond to Group A, whereas those on the right correspond to Group B. (1) Lips of the reticular groove; (2) reticulo-omasal orifice. (A) Reticulum from Group A. (B) Reticulum from Group B. (C) Detail of reticular cells in Group A. (D) Detail of reticular cells in Group B. (E) Higher magnification of reticular cells in Group A. (F) Higher magnification of reticular cells in Group B.

Macroscopic examination revealed that the reticular mucosa was organized into primary crests that anastomosed to form polygonal compartments, including square, rectangular, and hexagonal cells (Figure 2).

**Figure 3:**
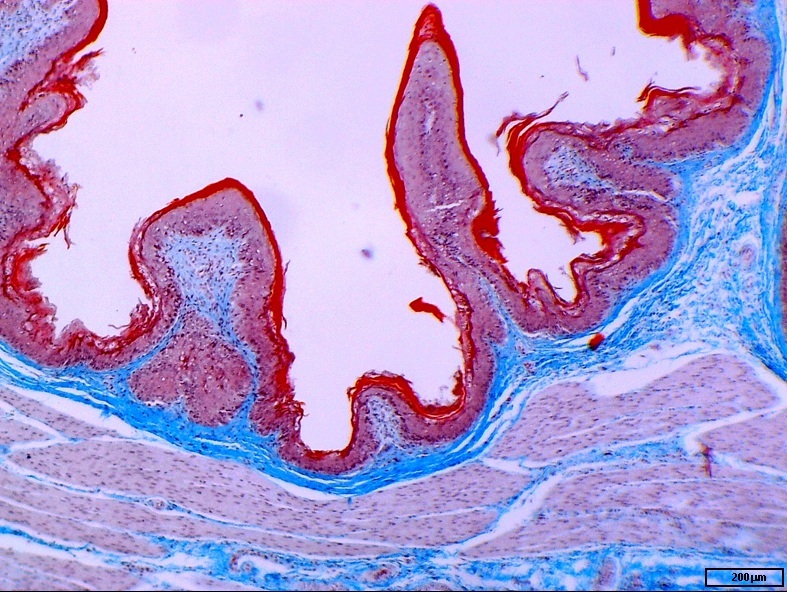
Optical microscopy images of the mid-region of the reticulum, showing papillary development and the keratin layer (Masson-Goldner trichrome). Group B. 4X.

Secondary and tertiary crests subdivided the primary cells in both groups, forming smaller compartments. The development of secondary and tertiary crests appeared more pronounced in Group B (Figures 2 and 3).

All primary crests bore well-defined papillae along their free margins, with smaller papillae present on lateral surfaces.

#### Regional variations in reticular cells

In the cardia-adjacent region, calves in Group B exhibited square and hexagonal cells, whereas Group A calves displayed square and rectangular cells.

In the mid-reticulum, Group B calves presented square, rectangular, and hexagonal cells, while Group A calves showed predominantly square and hexagonal cells.

Near the reticulo-omasal orifice, primary cells in Group A were mainly hexagonal, heptagonal, or octagonal, whereas Group B exhibited exclusively hexagonal cells (Figure 2).

#### Papillary development

**Figure 4:**
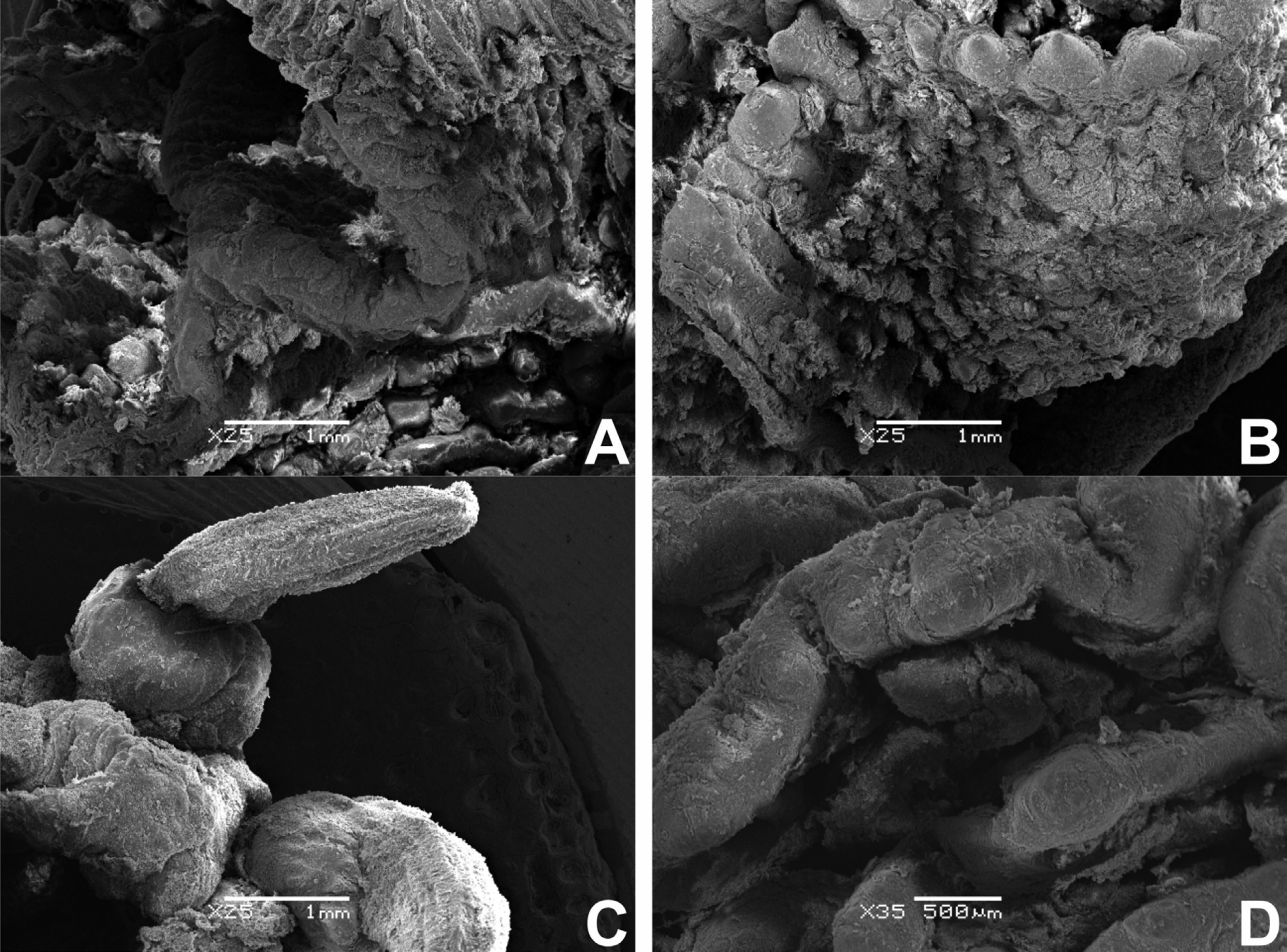
Scanning microscope images of the reticular epithelium of the different regions showing crest and papillae development. On the left are images from Group A and on the right are images from Group B. A, B and C 25X. D 35X. A and B- Mid region; C and D reticular groove.

In Group B, free-margin papillae were larger and more pointed than those in Group A (Figure 4). At the base of the reticular cells, elongated and pointed papillae were arranged at multiple levels and were more numerous and larger in Group B (Figures 2 and 4).

**Figure 5:**
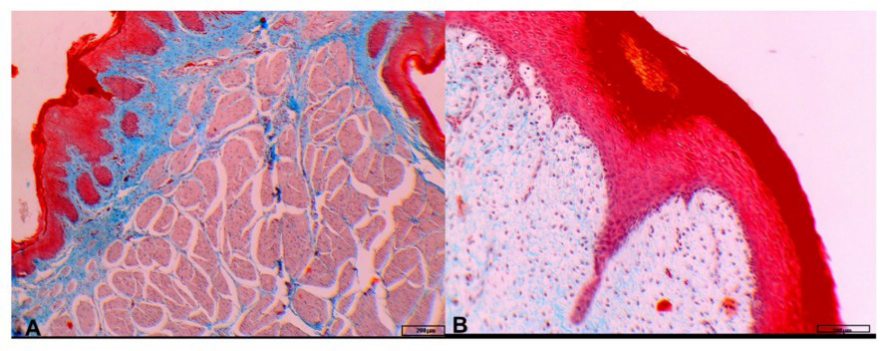
Optical microscopy images of the reticulum, showing a unguiculiform papillae and the keratin layer (Masson-Goldner trichrome).A- 4X. B- 10X.

Near the reticulo-omasal orifice, highly keratinized conical papillae (unguiculiform papillae) were observed. These structures were larger and more developed in Group B (Figures 4 c and 5).

#### Reticular groove

The reticular groove extended from the cardia to the reticulo-omasal orifice and continued as the omasal canal. Its right and left lips were thickened and protruding, with no significant differences in thickness between groups (Table 3).

**Figure 6:**
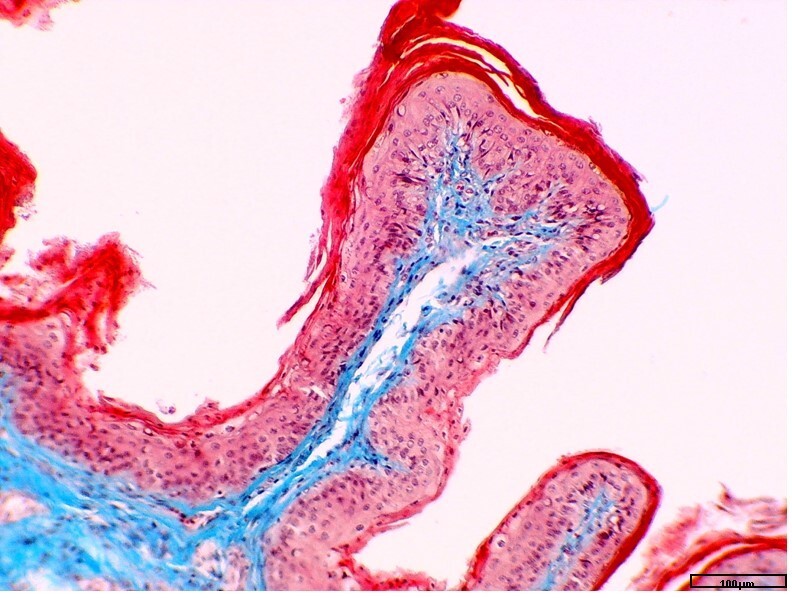
Optical microscopy images of the lips of the reticulum (Masson-Goldner trichrome). Group A. 10X.

The mucosa covering the lips exhibited a pronounced wrinkled pattern along the free margin and outer surfaces, whereas the inner surfaces and the base of the groove displayed a smooth, whitish mucosa with fine longitudinal crests (Figure 6).

## Discussion

This study is among the first to specifically examine reticular morphology in young Holstein calves receiving 8 L/day of milk replacer, supplemented with either starter feed (Group A) or alfalfa hay (Group B). Most previous research has treated the reticulo-rumen as a single structure, measuring combined weights and ruminal papillae, without focusing on the reticulum itself, including features such as reticular cells and crest architecture (Terler et al., 2023; Yohe et al., 2022).

By contrast, our approach emphasizes the distinct morphological characteristics of the reticulum, including primary, secondary, and tertiary crests, as well as unguiculiform papillae. While the scope of the study was limited to a short pre-weaning period and a relatively small experimental group, the results provide novel insights into how early-life solid feed composition influences reticular development.

From a functional perspective, the reticulum plays a critical role in digesta stratification, particle sorting, and regulation of passage through the reticulo-omasal orifice (Clauss et al., 2009; Okine et al., 1998). Structural features such as crest height, cell subdivision, and papillary development are directly associated with the efficiency of these processes and are known to differ markedly among ruminant species according to feeding strategy (Hofmann, 1973; Langer, 1988). The present findings suggest that similar adaptive responses can be elicited during early postnatal development through dietary manipulation, even within a single species.

Although no statistically significant differences were observed in gross reticulum weight or external dimensions between groups, histological and ultrastructural evaluations revealed greater structural complexity in forage-fed calves (Group B). These animals displayed more developed primary, secondary, and tertiary crests, larger papillae along the free margins, and more pronounced unguiculiform papillae.

These morphological traits are characteristic of grazing ruminants and have been associated with increased retention of fibrous particles and enhanced microbial fermentation (Clauss et al., 2009; Hofmann, 1973). In wild and domestic grazers, a higher degree of crest subdivision increases the effective surface area and promotes selective retention of larger particles, thereby slowing digesta passage and improving fiber utilization. The more advanced crest architecture observed in Group B calves may therefore reflect an early functional adaptation to a fiber-rich solid feed, despite the high liquid intake typical of intensive milk replacer programs.

The observed differences in papillary development further support this interpretation. Unguiculiform papillae, located near the reticulo-omasal orifice and reticular groove, have been described as playing a mechanical filtering role, limiting the transfer of coarse particles to the omasum (Teixeira et al., 2009). In the present study, these papillae were larger and more developed in calves receiving alfalfa hay, suggesting an enhanced capacity for selective digesta flow regulation.

This finding is particularly relevant given that, in young calves, the reticular groove and its associated structures are also involved in directing milk away from the forestomach during suckling (Dyce et al., 2012; Schummer et al., 1981). The concurrent development of filtering papillae and groove-associated structures may indicate that solid feed characteristics influence not only epithelial growth but also the functional maturation of flow-control mechanisms within the reticulum.

The present results align with those of previous studies demonstrating that the type and physical form of solid feed modulate forestomach development. Yohe et al. (2022) reported that Holstein calves fed high milk replacer allowances combined with starch-rich starter exhibited enhanced visceral tissue growth and ruminal papillae development. Similarly, Terler et al. (2023) showed that the inclusion of hay of different qualities alongside concentrate altered rumen histology and digestive tract development in young calves.

However, these studies primarily focused on the rumen or combined reticulo-rumen measurements and did not assess the reticulum as a distinct anatomical entity. Our findings extend this body of knowledge by demonstrating that dietary fiber specifically influences reticular micro- and macro-architecture, even when overall reticular mass remains unchanged. This supports the concept that qualitative morphological adaptations may precede measurable changes in organ weight or size during early development.

Mechanistically, the reduced structural complexity observed in starter-fed calves (Group A) may be explained by differences in both chemical and mechanical signaling pathways regulating epithelial growth. Concentrate-based diets increase ruminal production of short-chain fatty acids, particularly butyrate, which is known to stimulate epithelial proliferation and differentiation through mechanisms including modulation of gene transcription, and activation of growth-related pathways such as insulin-like growth factor-1 (IGF-1) signaling (Sakata & Tamate, 1979; Baldwin et al., 2004). Butyrate also enhances ruminal epithelial development by promoting cell cycle progression and angiogenesis (Baldwin et al., 2004). However, while these trophic effects are primarily described for the rumen, similar epithelial responses may occur in the reticulum given the shared stratified squamous epithelium of the reticulo-rumen complex.

In contrast, long-fiber forage provides substantial mechanical stimulation of the mucosa. Physical abrasion and distension of the reticular wall may activate mechanotransduction pathways involving integrins, focal adhesion kinase (FAK), and downstream effectors such as the YAP/TAZ signaling cascade, which are recognized regulators of epithelial proliferation and tissue architecture in mechanically responsive epithelia (Dupont et al., 2011; Ingber, 2006). Enhanced crest subdivision and papillary elongation in forage-fed calves (Group B) may therefore reflect the combined influence of mechanical strain–induced cytoskeletal remodeling and localized proliferative signaling. The comparatively lower physically effective fiber content of starter diets would provide less sustained mechanical input, potentially explaining the reduced architectural complexity observed in Group A despite adequate fermentative stimulation.

## Conclusion

Overall, these results underscore the importance of considering the reticulum as a distinct anatomical and functional unit in studies of early-life nutrition, rather than subsuming it within the reticulo-rumen. They also confirm that the inclusion of dietary fiber during the pre-weaning period induces measurable morphological adaptations in the reticulum, which may contribute to improved digesta handling and fermentation efficiency later in life. Nevertheless, the interpretation of these findings must explicitly acknowledge the limited sample size used for detailed microscopic and ultrastructural analyses (n = 2 calves per group). Although consistent morphological patterns were observed between individuals within each treatment, this small number substantially limits statistical power and increases the risk that subtle inter-individual variability was not captured. Consequently, histological and ultrastructural observations should be interpreted as exploratory and hypothesis-generating rather than definitive. Future studies using larger cohorts and quantitative morphometric approaches are necessary to confirm the reproducibility and functional significance of the structural differences described herein. In addition, the relatively short duration of the experimental period restricts extrapolation to post-weaning development and long-term productive outcomes.
